# Integration of *Arabidopsis thaliana *stress-related transcript profiles, promoter structures, and cell-specific expression

**DOI:** 10.1186/gb-2007-8-4-r49

**Published:** 2007-04-04

**Authors:** Shisong Ma, Hans J Bohnert

**Affiliations:** 1 Physiological and Molecular Plant Biology Graduate Program, University of Illinois at Urbana-Champaign, Urbana, IL 61801, USA; 2 Department of Plant Biology, University of Illinois at Urbana-Champaign, Urbana, IL 61801, USA; 3 Department of Crop Sciences, University of Illinois at Urbana-Champaign, Urbana, IL 61801, USA

## Abstract

**Background:**

*Arabidopsis thaliana *transcript profiles indicate effects of abiotic and biotic stresses and tissue-specific and cell-specific gene expression. Organizing these datasets could reveal the structure and mechanisms of responses and crosstalk between pathways, and in which cells the plants perceive, signal, respond to, and integrate environmental inputs.

**Results:**

We clustered *Arabidopsis *transcript profiles for various treatments, including abiotic, biotic, and chemical stresses. Ubiquitous stress responses in *Arabidopsis*, similar to those of fungi and animals, employ genes in pathways related to mitogen-activated protein kinases, *Snf1*-related kinases, vesicle transport, mitochondrial functions, and the transcription machinery. Induced responses to stresses are attributed to genes whose promoters are characterized by a small number of regulatory motifs, although secondary motifs were also apparent. Most genes that are downregulated by stresses exhibited distinct tissue-specific expression patterns and appear to be under developmental regulation. The abscisic acid-dependent transcriptome is delineated in the cluster structure, whereas functions that are dependent on reactive oxygen species are widely distributed, indicating that evolutionary pressures confer distinct responses to different stresses in time and space. Cell lineages in roots express stress-responsive genes at different levels. Intersections of stress-responsive and cell-specific profiles identified cell lineages affected by abiotic stress.

**Conclusion:**

By analyzing the stress-dependent expression profile, we define a common stress transcriptome that apparently represents universal cell-level stress responses. Combining stress-dependent and tissue-specific and cell-specific expression profiles, and *Arabidopsis *5'-regulatory DNA sequences, we confirm known stress-related 5' *cis*-elements on a genome-wide scale, identify secondary motifs, and place the stress response within the context of tissues and cell lineages in the *Arabidopsis *root.

## Abstract 2

The integration of stress-dependent, tissue- and cell-specific expression profiles and 5'-regulatory sequence motif analysis defines a common stress transcriptome, identifies major motifs for stress response, and places stress response in the context of tissue and cell lineages in the *Arabidopsis *root.

## Background

Knowledge about responses of the model plant *Arabidopsis thaliana *to abiotic or biotic stresses has accumulated during the past decade, based on large-scale mutant analyses and genome-wide transcript profiles. In particular, random mutagenesis combined with cell-specific or treatment-specific reporter gene expression has identified many players in the stress response, whereas microarray-based observations have revealed transcriptional responses to stresses on a genome-wide scale [1-4]. However, most analyses have been restricted to individual genes or treatments. Plant-specific databases, such as The *Arabidopsis *Information Resource (TAIR), Genevestigator, and the Nottingham Arabidopsis Stock Centre (NASC), have begun to collect data from various sources and merge them with genome sequence-based features [5-8]; however, the data typically exist in isolation. Integrating these diverse datasets remains a significant challenge in the assembly of a unifying picture of plant responses to environmental effects. For this purpose, various tools have been developed, such as MapMan and STKE (Signal Transduction Knowledge Environment), which begin to link individual genes to pathways or coregulation circuits [9,10]. Here, we present an alternative approach to integrating different datasets related to plant stress responses.

In *Arabidopsis*, as in all organisms, a variety of stress factors that disturb homeostatic conditions bring about ubiquitous as well as distinct responses at the transcription level. Identification of ubiquitous, cell autonomous responses is based on monitoring the status of macromolecules in cells, gauging DNA damage, protein degradation, or lipid membrane integrity, and eliciting pathways that carry out repair functions [11]. The degree of damage will trigger this common response, which must be distinguished from a set of reactions that recognize and respond to specific stress conditions. Identifying the genes that determine the specific responses and then separating them into distinct groups, functional categories, and pathways is an important task that must be undertaken if we are to elucidate how plants sense and recognize the environment, and then embark upon a meaningful defense that will alleviate the stress condition. The approach presented here aims to define the distinction between ubiquitous and specific stress response categories. Very few transcript profiling studies, which did not include the majority of the *Arabidopsis *genes, have addressed specificity and crosstalk of different stress treatments [1,3,4].

Control over gene expression is in part determined by motifs, *cis*-elements, within the promoter sequence of regulated genes. In plants, distinct motifs have been correlated with responses to individual treatments, resulting in discovery of a number of motifs related to stress responses and developmental or organ-specific regulation. Among these motifs, those responding to light and osmotic and cold stress treatments have been analyzed most intensely [12,13]. Databases dedicated to plant promoter motifs have been established, based on motif identification in single or, at most, a few genes [14,15]. How their competence in regulating gene expression is mirrored at the genome level has not been tested.

Here, we applied the fuzzy k-means clustering method [16] to publicly available microarray data from the AtGenExpression project to compare the response of *Arabidopsis *to a variety of abiotic and biotic stresses that disturb homeostatic conditions [17]. The results revealed common as well as distinct pathways that govern changes in the expression of induced and repressed genes in response to various treatments. Based on the collection of motifs in the Plant *cis*-acting Regulatory DNA Elements (PLACE) database [14], clusters of coregulated genes were screened for over-represented *cis*-elements within their promoters. In addition, gene expression profiles identifying cell lineages in *Arabidopsis *roots were used to correlate the cell type-specific response to various stresses in the root [18,19]. Integration of information from previously unconnected databases provided surprising insights about genes and pathways that classify the evolutionarily conserved cell-based common stress response, and the divergent pathways that organize abscisic acid (ABA)-dependent and ABA-independent reactions to stress in a tissue-specific manner.

## Results and discussion

**Figure 1:**
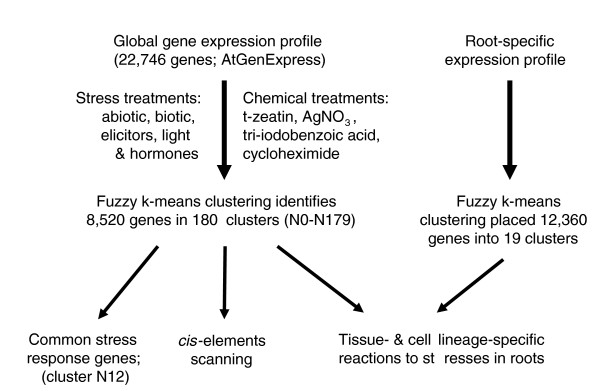
Strategies to identify of *Arabidopsis *stress-regulated and tissue-regulated genes.

An analysis of the *Arabidopsis *abiotic and biotic transcriptome is presented in four sections (Figure 1). First, the overall clustering pattern for 22,746 probes in response to different environmental and chemical stress conditions was analyzed. This was followed by analysis of a 'common stress transcriptome', which unites genes that respond to any deviation from homeostasis. Then, an analysis of 5'-motifs defined promoter structures - *cis*-elements - that are characteristic for individual clusters of stress-responsive genes, focusing on clusters containing induced genes (2,715 genes in total) and on the few large clusters (5,998 genes) containing stress-repressed genes. Finally, cell-specific and tissue-specific responses to a variety of stresses were determined by integrating the clusters defining stress specificity with the gene expression map established for the *Arabidopsis *root [19]. This analysis provided intersections between stress and tissue or cell specificity.

### Clustering of different stress response categories

**Figure 2:**
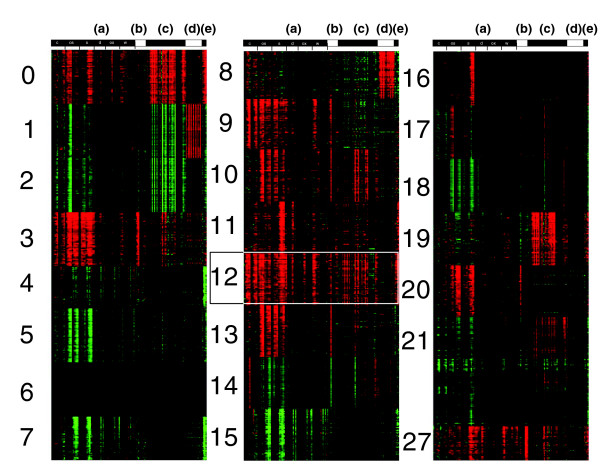
Clustering of genes in the *Arabidopsis *transcriptome. Out of 22,746 genes, 10,671 genes exhibited significant membership values in 180 clusters. The 17 most populated clusters include 7,039 genes (66% of total). Rows represent individual genes; columns (from left to right, as listed below) represent treatment conditions. A total of 180 clusters emerged. Outlined is cluster 12 (216 genes) including genes that responded to all stress treatment conditions (see Additional data files). **(a) **Time course experiments include cold (12 time points), osmotic (12), salt (12), drought (12), oxidative (12), and wounding (14) treatments. **(b) **Hormone treatments include ABA (3), ACC (3) and MeJA (3). **(c) **Biotic stress treatments include bacteria-derived elicitors (12), *Pseudomonas syringae *pt. tomato (Pst) DC300 (3), Pst avrRPM1 (3), Pst DC3000hrcC- (3), *P. syringae *pv. phaseolicola (3), *Erysyphe oromoti *(7), *Phytophtera infestans *(3), *P. syringae *ES4325 avrRPT2 (5), and *P. syingae *ES4325 (5). **(d) **Different light conditions (14). **(e) **Chemical treatments included t-zeatin, tri-iodobenzoic acid, AgNO_3_, and cycloheximide.

The fuzzy k-means clustering method [16,20,21] was applied to the probe set (22,746 in total) printed on Affymetrix *Arabidopsis *ATH1 chips, which corresponded to about 22,400 genes. In the following analysis, we treated each probe set as a gene. The external conditions selected included treatments with a variety of biotic and abiotic stresses included in AtGenExpress [17], as outlined in a previous analysis that focused on a subset of salt-responsive genes [21]. Additionally included were results for different light conditions and exposures of plants to chemicals and growth regulators such as t-zeatin, tri-iodobenzoic acid, AgNO_3_, and cycloheximide. The chemical treatments were included because we expected them to add additional power of resolution to the analysis. Considering the large number of genes to be analyzed, fuzzy k-means clustering was conducted initially with a large centroid parameter (k = 320). Subsequently, 10,490 genes with significant membership values emerged from the dataset, which, with the cutoff set at a membership value of 0.035, most parsimoniously assembled into 180 clusters. The composition of 28 clusters (N0 to N27) is shown in Figure 2 and the entire set is included in the Additional data files 2 and 3.

The 'limma' statistical program was applied to the Affymetrix dataset to identify differentially regulated genes [22]. Of the 22,746 probe sets, 14,015 were differentially expressed in at least one condition (*P *< 10^-15^). Among the 10,490 significant genes included in the clustering analysis, 8,520 were differentially expressed and 1,970 were not significantly regulated. This nonregulated category includes 879 (out of 884) and 119 (out of 131) genes from clusters N6 and N53, respectively. Genes in cluster N6 were not regulated under most conditions, whereas genes in cluster N53 exhibited a very small induction in osmotically-stressed roots only (see Additional data file 4). The separation of clusters N6 and N53 reflects the discriminative power of fuzzy k-means clustering and sensitivity in identifying even minute differences in expression patterns. The remaining nonregulated genes were mainly found in downregulated clusters. In the following analysis of common stress responses and promoter motifs, we focus our attention on the 8,520 differentially expressed genes.

The majority of these 8,520 genes was concentrated in a few large clusters. The most highly populated 15 clusters, each including more than 100 genes, totaled 5,478 or more than 60% of all significantly clustered transcripts. The largest clusters, namely N0, N2, N5, N18, included 699, 1,206, 705, and 430 genes, respectively. ABA, which acts as an important signaling molecule under a variety of different stress conditions, was implicated in and induced the expression of genes in clusters N3, N9, N10, N12, N13 and N20, whereas genes in clusters N0, N11, N16, N19 and N28 did not respond to ABA (Figure 2). Genes in clusters N1 and N8 were induced by light, and those in cluster N1 were additionally repressed in response to biotic stress treatments. Genes in cluster N27 were induced by jasmonic acid (JA) treatment, as well as by salt and wounding stresses. Large clusters in which gene expression was generally repressed by environmental stresses included N2, N4, N5, N7, N15, and N18. All genes are identified in the Additional data files.

#### The 'universal stress response transcriptome': cluster N12

**Figure 3:**
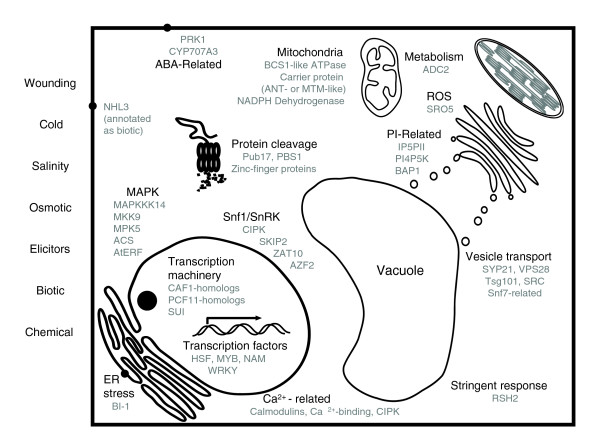
Diagram of common stress response pathway genes. Representation of genes with known functions in clusters that respond to most stresses in cluster N12. Genes are identified by name or Gene Ontology assignment (see Additional data file 5).

The 197 genes in cluster N12 (Figure 2) are induced by a broad range of diverse stress conditions: cold, osmotic, salinity, wounding, and biotic stresses (including treatments with elicitors). The 'limma' analysis indicated that approximately 80% of these genes were significantly regulated under all treatment conditions, whereas the rest of the included genes were marginally regulated in one (mostly the wounding treatment) but significantly regulated in all other conditions (*P *< 0.01; Table 1; Additional data file 5). They appear to represent a common or universal stress response transcriptome because most of these genes are conserved among plants, animals and fungi, and are stress regulated in all organisms, with the inclusion of a few genes related to the plant-specific hormones ABA and JA (Figure 3 and Table 1). Several Gene Ontology (GO) categories were enriched among these genes: GO:0009611 (response to wounding), GO:0009613 (response to pest, pathogen, or parasite), GO:0006970 (response to osmotic stress), GO:0009737 (response to ABA stimulus), GO:0009651 (response to salt stress), GO:0009723 (response to ethylene stimulus), GO:0009751 (response to salicylic acid stimulus), GO:0009753 (response to JA stimulus), GO:0050832 (defense response to fungi), GO:0006839 (mitochondrial transport), and GO:008270 (zinc ion binding). Signaling pathways related to mitogen-activated protein kinase (MAPK), calcium, reactive oxygen species (ROS), phospholipids, apoptosis, and protein degradation were induced. Equally, part of this cluster of genes that generally are upregulated by stress is functionally related to vesicle transport and mitochondrial functions. N12 included induced genes that had previously been identified as related to or specific for biotic stresses, but these were also induced by abiotic stresses, and *vice versa*. Past restrictions in the scope of analyses, which typically focused on single treatment conditions, and the resulting problem of annotation stringency did not compromise the fuzzy k-means clustering analysis. We discuss these universal stress response genes by organizing them into different pathways (Figure 3).

**Table 1:** Selected common stress response genes

Affymetrix probe	AGI	Annotation	Membership value
257053_at	At3g15210	ATERF-4	0.273508
261470_at	At1g28370	ERF/AP2 transcription factor	0.162002
248799_at	At5g47230	ATERF-5	0.086731
252214_at*	At3g50260	ERF/AP2 transcription factor	0.083494
245250_at	At4g17490	ATERF-6	0.063712
248448_at	At5g51190	ERF/AP2 transcription factor	0.044611
254926_at	At4g11280	ACS6	0.109595
266832_at	At2g30040	MAPKKK14	0.054407
245731_at	At1g73500	ATMKK9	0.165439
254924_at	At4g11330	ATMPK5	0.060749

247033_at	At5g67250	SKIP2	0.052666
255872_at	At2g30360	CIPK11	0.093386
261648_at	At1g27730	ZAT10	0.458157
257022_at	At3g19580	AZF2	0.194905

248833_at	At5g47120	Bax inhibitor-1, AtBI-1	0.048683

246453_at	At5g16830	SYP21	0.089816
254422_at	At4g21560	VPS28 family protein	0.081642
264655_at	At1g09070	SRC2	0.119115
256238_at	At3g12400	tumour susceptibility gene 101 (TSG101) family protein	0.051677
265375_at	At2g06530	SNF7 family protein	0.125775
262367_at*	At1g73030	SNF7 family protein	0.037115
247204_at	At5g64990	Ras-related GTP-binding protein, putative	0.048757

260915_at	At1g02660	lipase class 3 family protein	0.100258
254707_at	At4g18010	inositol polyphosphate 5-phosphatase II (IP5PII)	0.056767
251336_at	At3g61190	BON1-associated protein 1 (BAP1)	0.152337
262540_at	At1g34260	phosphatidylinositol-4-phosphate 5-kinase family protein	0.054866

247431_at*	At5g62520	SRO5, similarity to RCD1 but without the WWE domain	0.048374
247655_at	At5g59820	zinc finger protein ZAT12	0.286685

259879_at*	At1g76650	calcium-binding EF hand family protein	0.09479
266371_at	At2g41410	putative calmodulin	0.072498
259137_at	At3g10300	calcium-binding EF hand family protein	0.06951
247426_at	At5g62570	calmodulin-binding protein	0.068879
247137_at	At5g66210	CPK28, calcium-dependent protein kinase	0.067785
251636_at	At3g57530	CPK32, calcium-dependent protein kinase	0.06706
253284_at	At4g34150	C2 domain-containing protein	0.056923
253915_at	At4g27280	calcium-binding EF hand family protein	0.051136
265460_at	At2g46600	calcium-binding protein, putative	0.038761

249928_at	At5g22250	similar CCR4-NOT transcription complex, subunit 7, CAF1	0.136686
248146_at	At5g54940	eukaryotic translation initiation factor SUI1	0.090271
256356_s_at	At1g66500	similar to Pre-mRNA cleavage complex II protein Pcf11	0.102701
255742_at	At1g25560	AP2 domain-containing transcription factor	0.039118
245247_at	At4g17230	scarecrow-like transcription factor 13, SCL13	0.251161
246987_at	At5g67300	myb family transcription factor	0.096819
265359_at	At2g16720	myb family transcription factor, MYB7	0.068213
246253_at*	At4g37260	myb family transcription factor, MYB73	0.046837
253219_at	At4g34990	myb family transcription factor, MYB32	0.03538
247351_at	At5g63790	no apical meristem (NAM) family protein	0.159799
252278_at	At3g49530	no apical meristem (NAM) family protein	0.127213
249746_at	At5g24590	turnip crinkle virus-interacting protein, with NAM domain	0.087334
261892_at	At1g80840	WRKY family transcription factor, WRKY40	0.186156
267028_at	At2g38470	WRKY family transcription factor, WRKY33	0.122218
267246_at	At2g30250	WRKY family transcription factor, WRKY25	0.069374
253535_at*	At4g31550	WRKY family transcription factor, WRKY11	0.039245
253485_at	At4g31800	WRKY family transcription factor, WRKY18	0.037623
247509_at	At5g62020	Heat Stress Transcription Factor, At-HSFB2A	0.110848
254592_at*	At4g18880	Heat Stress Transcription Factor, At-HSFA4A	0.084085
259992_at*	At1g67970	Heat Stress Transcription Factor, At-HSFA8	0.069577

255259_at	At4g05020	NADH dehydrogenase-related	0.089839
254120_at	At4g24570	mitochondrial substrate carrier family protein	0.111919
250335_at	At5g11650	hydrolase, alpha/beta fold family protein	0.111254
252131_at	At3g50930	AAA-type ATPase family protein	0.105058
250062_at	At5g17760	AAA-type ATPase family protein	0.052915
265450_at*	At2g46620	AAA-type ATPase family protein	0.05194
253323_at	At4g33920	PP2c familiy protein	0.10342
253824_at	At4g27940	mitochondrial substrate carrier family protein	0.075698
246870_at	At5g26030	ferrochelatase I	0.089846
264000_at	At2g22500	mitochondrial substrate carrier family protein	0.308636
246779_at	At5g27520	mitochondrial substrate carrier family protein	0.077536
251757_at	At3g55640	mitochondrial substrate carrier family protein	0.040783

260345_at*	At1g69270	leucine-reich repreat family protein, RPK1	0.061161
248964_at	At5g45340	P450 CYP707A3	0.050208

253203_at	At4g34710	arginine decarboxylase, ADC2	0.099102

258207_at	At3g14050	RelA/Spot protein, RSH2	0.177302

250676_at	At5g06320	harpin-induced family protein, NHL3	0.119862
259826_at	At1g29340	PUB17, an E3 ubiquitin ligase	0.069476
267411_at	At2g34930	disease resistance family protein, similar to Cf-2.1	0.043403
245986_at*	At5g13160	protein kinase PBS1	0.06912

Included are genes from the common stress response cluster N12. Membership indicates the probe membership value associated with centroid N12. Asterisks identify genes that are significantly regulated in all but one treatment condition (see results).

#### MAPK pathways

Several MAPK pathways, organized into signaling cascades, are conserved in eukaryotic organism [23,24]. In *Saccharomyces cerevisiae*, for example, the high osmolarity glycerol (HOG) signaling pathway is responsible for osmotic stress sensing [25,26]. The *Arabidopsis AtHK1, MEK1, MPK4*, and *MPK6 *can complement yeast deletion mutants of the HOG pathways. Other examples of plant MAPKs are alfalfa stress-induced MAPK (*SIMK*), tobacco salicylic acid-induced protein kinase (*SIPK*), wound-induced protein kinase (*WIPK*), and *Nicotiana *Fus-3-like kinase6 (*Ntf6*).

Among common genes that are upregulated by stress, several MAPK components were identified: *MPK5, MKK9*, and *MAPKKK14*. The MAPK pathway has been suggested to be involved in ethylene signaling [27-29]. Included among ubiquitous stress-regulated genes is also *ACS6*, encoding the rate-limiting enzyme of ethylene biosynthesis and a substrate for MPK6 [30], together with six ERF/AP2 transcription factors (AtERF). This implicates the ethylene signaling-mediated engagement of a subset of the MAPK family as a component of the common stress response.

However, the ethylene response transcriptome is not strictly clustered in the stress transcriptome, notwithstanding its importance in developmental processes such as fruit ripening. Incorporating the results from a study that measured transcript changes in *Arabidopsis *Col-0 wild-type [31] into the cluster structure obtained by fuzzy k-means, the significantly ethylene-regulated genes identified in the study were located in a large number of different clusters.

#### *Snf1/AMPK/SnRK*

The yeast Snf1 protein kinase and the mammalian AMP-activated protein kinase act as metabolic sensors that monitor cellular AMP and ATP levels. Activation increases the ATP:AMP ratio. Snf4 is part of the Snf1 protein kinase complex. In higher plants, they are involved in response to environmental or nutritional stress. Related common stress-induced genes were *CIPK11 *(encoding a Snf1-related protein kinase that is similar to SOS2, a protein kinase that is involved in plant salinity stress responses) [32], *SKIP2 *(a conserved SCF ubiquitin ligase subunit that interacts with SnRKs), and *AZF2 *and *ZAT10 *(C2H2 zinc finger proteins) [33]. Both *AZF2 *and *ZAT10 *suppressed the *Snf4 *deficiency in yeast and function as transcription repressors in *Arabidopsis *[33,34]. ZAT10 can activate salt stress tolerance, controlled in yeast by MSN2 and MSN4 factors, and ZAT10 can repress the expression of the plant stress gene *RD29A *[35]. Several Snf1-related genes appeared in stress-induced clusters other than N12 as well, suggesting functions that are specific for particular stress conditions (data not shown).

#### Bax inhibitor 1: endoplasmic reticulum stress

The Bax-inhibitor 1 (BI-1) is an endoplasmic reticulum (ER) protein that suppresses cell death induced by ER stress in both animal and plant cells. It can inhibit the activation of Bax and its translocation to mitochondria, and suppresses the activation of caspase, and functions in reducing calcium release from the ER [36]. In *Arabidopsis*, Bax over-expression causes ROS accumulation and cell death, and BI-1 attenuates the cell death effect without affecting production of ROS [37,38]. It alleviated cell death caused by biotic and abiotic stresses [39]. *BI-1 *(At5g47120), one of three genes in *Arabidopsis *with this sequence signature, was induced by several other stresses in a specific manner as well, and appears to represent a signature gene and protein of the common stress response cluster.

#### Vesicle transport

Although mechanisms of vesicle transport have been studied extensively, little is known about regulation in response to stress. A plant vesicle-related protein, AtVAMP, when ectopically expressed, can suppress Bax-induced apoptosis in yeast, possibly by improving membrane repair [40]. The over-expression of *AtRab7*, a gene that is involved in regulation of vesicle trafficking, increased endocytosis in roots, as well as salt and osmotic stress tolerance [41]. This indicates the importance of regulated vesicle trafficking for acquisition of stress tolerance.

Several genes related to trafficking from endosomes to central vacuoles were placed into N12. They are *SYP21*, *Vps28*-related, *Tsg101*-related, *SRC2*, Ras-related GTPase, and genes for two Snf7 family proteins. In roots, the *Tsg101*-related and *Vps28*-related genes, as well as *SYP21 *and one gene encoding a Snf7-like protein are specifically expressed in the endodermis of the root hair zone.

#### Phospholipid signaling

A multitude of signaling molecules is generated from membrane phospholipids. Their involvement in osmotic stress responses has been demonstrated. Several related genes are induced, such as encoding inositol polyphosphate 5-phosphatase II, FYVE domain-containing phosphatidylinositol-4-phosphate 5-kinase (PI4P5K), and lipase class 3 family proteins. PI4P5K leads to the synthesis of PI4,5P2. Mutations in the offsetting phosphatase gene, *SAC9*, lead to over-accumulation of PI4,5P2 and constitutive expression of stress-response pathways [42,43]. The product of the *BAP1 *gene, which is also upregulated, interacts with BON1, a protein with two C2 domains that binds to phospholipids. Together, *BAP1 *and *BON1 *control plant growth homeostasis [44].

#### Reactive oxygen species

ROS have been associated with stress sensing and signaling, but have emerged more recently as important, general signals [45-47]. Irrespective of their ubiquitous presence, ROS that derive from different stimuli appear to be recognized as specific, indicating that a number of different signal mediators must exist. We suggest that cluster 12 identifies the evolutionarily conserved set of these genes. *SRO5 *is a gene that controls ROS in plants, which is upregulated by various stresses. *SRO5 *transcript expression overlaps partially with that of *P5CDH *mRNA. The induction of *SRO5 *leads to production of a 24-nucleotide nat-siRNA that guides cleavage of P5CDH mRNA, resulting in regulated proline levels [48]. Additionally, *ZAT12*, and possibly *ZAT10 *of the Snf1 pathway, also participate in ROS signaling transduction [46].

#### Calcium

Multiple calcium-related functions are induced by stresses. Among them is a SOS2-like protein kinase, namely CIPK11. However, little is known about the other genes in this group, including two calmodulins, three calcium-binding proteins, and three calcium-dependent kinases. These calcium-related genes cannot be organized into a pathway-like structure, in part because of the lack of detailed experimental evidence and also based on the multiplicity of functions that are channeled through calcium-binding proteins.

#### The transcription machinery and transcription factors

CCR4 and CCR4-associated factor 1 (*CAF1*) are critical for mRNA turnover in yeast [49]. Pcf11 is an mRNA 3'-end processing factor and binds the carboxyl-terminal domain of the largest subunit of RNA polymerase II [50]. Both *CAF1 *and *Pcf11 *have their *Arabidopsis *homologs upregulated by different stresses, indicating a role for control over mRNA processing and degradation. Another upregulated gene is the eukaryotic translation initiation factor *SUI1*. Other examples are *AZF2 *and *ZAT10*, which encode transcription repressors.

Stress-related transcription factors were also among the common stress response genes, including five WRKY family members, four Myb, three HSF, three NAM and two AP2, and the transcription factor SCL13. Included are WRKY18 and WRKY40, which physically interact with both overlapping and antagonistic roles in pathogen responses [51]. WRKY25 and WRKY33 are substrates of MKS1, which itself is a substrate of MPK4 and regulates plant defense reactions [52]. WRKY33 is also required for resistance to necrotrophic fungal pathogens [53]. WRKY11 interacts with calmodulin and acts as a negative regulator of basal resistance in *Arabidopsis *[54]. *SCL13 *has been shown to function in light signaling [55]. These WRKYs function in resistance to necrotrophic but not biotrophic pathogens, whereas necrotrophic damage is more closely related to the physical damage caused by abiotic stresses, as also reflected in the cluster structures. Little information is available for other transcription factors in cluster N12, although several isoforms of Myb, NAM, HSF, and AP2 not included in N12 have been associated before with stress response pathways.

#### Mitochondrial functions

Among the genes upregulated by many stress treatments, several are localized to mitochondria. They are three BCS1-like ATPases (which could function as chaperones, whose yeast homologs are required for cytochrome bc[1] complex assembly), two DIC1-like, one ANT1-like, one MTM1-like, and one other mitochondrial substrate carrier family protein. Furthermore, a ferrochelatase I gene, an NADH dehydrogenase-related gene, and a PP2C are part of this group. Also upregulated here was the Bax-inhibitor 1 gene. To appreciate their precise functions in plants, more studies are required.

#### ABA-related: *RPK1 *and *CYP707A3*

Among the common stress response genes were two ABA-related genes, *RPK1 *and *CYP707A3*. *RPK1 *encodes a leucine-rich repeat receptor-like kinase 1, a membrane-bound regulator of ABA early signaling [56]. The *rpk1 *mutant exhibited decreased sensitivity to ABA, and over-expression resulted in hypersensitivity. *CYP707A3 *encodes a cytochrome P450 protein catalyzing ABA 8'-hydroxylation and catabolism. Its knockout mutant exhibited exaggerated ABA-inducible gene expression and enhanced drought tolerance, whereas over-expression was associated with growth retardation by ABA and increased transpiration [57].

#### ADC2, a rate-limiting enzyme in polyamine (PA) biosynthesis

ADC genes are essential for polyamine (PA) production. Over-expression of *ADC2 *led to GA-deficient plants and accumulation of putrescine, a phenotype reversed by GA3 [58]. The null mutant *adc2-1 *was sensitive to salt stress, but could be rescued by external putrescine [59]. *ADC2 *is among the common stress response genes.

#### RelA/SpoT, RSH2, and the 'stringent response' in bacteria

The stringent response is crucial for stress adaptation in bacteria, mediated by the production of the nucleotide guanosine-3',5'-(bis-)pyrophosphate (ppGpp). *RelA *and *SpoT *encode bacterial enzymes for ppGpp synthesis. *RSH *is the higher plant homolog of this RelA/SpoT protein [60,61].

#### *NHL3*, *PBS1*, and *PUB17*

These genes function in resistance to the bacterial pathogen *Pseudomonas syringae *pv. tomato DC3000 carrying avirulence proteins [51,62,63], and they - as identified here - were also induced by various abiotic stresses. Interestingly, *NHL3 *over-expression in *Arabidopsis *enhances resistance to the virulent *Pseudomonas syringae *pv. tomato DC3000, without an increase in PR gene expression or H_2_O_2 _accumulation [64]. *PBS1 *and *RPS5 *are required for avrPphB mediated *Pseudomonas syringae *resistance in *Arabidopsis*. AvrPphB can proteolytically cleave PBS1, which is required for RPS5-mediated resistance [65]. PUB17 is a U-box ARMADILLO repeat E3-ligase, which regulates cell death and defense [66]. Another disease resistance family protein, similar to Cf-2.1 (At2g34930), is also upregulated by various stresses. Its null mutant was particularly susceptible to fungus attack [67]. The inclusion of these genes in cluster N12 suggests their function in common mechanisms that counter both abiotic and biotic stresses.

#### Genes with unknown or unclear functions

An additional 120 genes are included in the common stress response cluster (ST3). In part, their functions are known by specific activities (for example, trehalose-6-phosphate phosphatase), whereas most are identified only by domain identifiers (for example, protease-associated or thioredoxin family-related), or their functions are not clear or completely unknown. The group included transcripts for 19 zinc-finger family proteins, five protein kinases, four protein phosphatases, a number of glycosyl hydrolases, thioredoxins, cytochromes P450, and hormone-responsive functions, mostly annotated according to similarity criteria, and 40 expressed proteins without any annotation. Among the genes that lack annotation, the majority is most strongly induced by conditions that affect redox homeostasis and ROS responses, in particular treatments with ozone, H_3_BO_3_, H_2_O_2_, AgNO_3_, hypoxia, and triiodobenzoic acid (an inhibitor of polar auxin transport; Genevestigator dataset [8]).

The high correlation of genes in cluster N12 with experimentally verified or alleged functions in a wide variety of stress conditions in species across all kingdoms suggests that the functions identified by this cluster categorize the basic stress response transcriptome (Figure 3). By their nature, these functions appear to identify ubiquitous cellular stress defense programs in all organisms, whereas pathways that integrate stress responses at the organ or organism levels may be based on programs that diverged during evolution. Conceivably, reverse genetics will determine the functions of little understood and completely unknown genes in N12, and provide a clear separation of these genes from pathways that are specific to individual stress conditions. The common stress response genes epitomize components of crosstalk between biotic and abiotic stress response mechanisms by identifying genes such as WRKY transcription factors, *NHL3*, and *PUB17*. Indeed, the *Arabidopsis *mutant *bos1 *exhibited compromised resistance to the pathogen *Botrytis cinerea *and reduced tolerance to drought, high salinity, and oxidative stress [68].

### Identification and analysis of regulatory motifs

Other clusters (Figure 2; ST1) separated the data into distinct groups, with groups of upregulated or downregulated genes with various groupings indicating dependence or independence of the action of hormones (ABA, ethylene, JA). Generally, all clusters included many genes with unknown functions but also a variable number of genes for which a relationship with a specific stress has been documented. One task was to analyze correlations between stress clusters and the presence and nature of regulatory motifs in their promoters.

We analyzed *cis*-elements, which are conserved motifs in the 5'-region of genes with a key role in assembling the transcription machinery. Extracted from the genome sequence were 1,000 base pairs upstream of the translation initiation codon, and genes in each cluster were scanned for motifs listed in the PLACE database [14]. The occurrence of these motifs was compared with their frequency among all promoters in the genome. A *P *value was then calculated for every motif and cluster combination, based on the hypergeometric distribution [69]. We considered motifs with *P *values lower than 10^-4 ^to be significantly over-represented. Listed in Table 2, and justified below, are motifs that have been identified.

**Table 2:** Promoter motifs in different clusters

**Motif**		**Cluster No.**	**Distribution**		***P *value**
			
**Name**	**Sequence**		**Genome**	**Cluster**	
WBBOXPCWRKY1	TTTGACT	N0	8004/31128	301/699	2.06 × e^-23^
	ACGCG*	N0	6880/31128	230/699	3.40 × e^-11^
HSF	RGAAnnTTC	N0	8380/31128	260/699	1.95 × e^-09^

ACGTABREMOTIFA2OSEM	ACGTGKC	N1	4552/31128	118/343	5.43 × e^-20^
ABREATCONSENSUS	YACGTGGC	N1	1470/31128	56/343	8.08 × e^-16^
IBOXCORENT	GATAAGR	N1	8237/31128	134/343	2.43 × e^-07^
SORLIP2AT	GGGCC	N1	10199/31128	145/343	1.44 × e^-04^
	RACCACAR*	N1	4187/31128	74/343	2.47 × e^-05^

IBOXCORENT	GATAAGR	N2	8237/31128	414/1206	9.99 × e^-10^
SORLIP2AT	GGGCC	N2	10199/31128	495/1206	1.06 × e^-09^
UP1ATMSD	GGCCCAWWW	N2	4460/31128	242/1206	3.53 × e^-08^
SORLIP5AT	GAGTGAG	N2	3015/31128	169/1206	9.47 × e^-07^

ACGTABREMOTIFA2OSEM	ACGTGKC	N3	4552/31128	98/154	1.09 × e^-43^
ABRELATERD1	ACGTG	N3	16971/31128	139/154	4.99 × e^-22^
BOXIIPCCHS	ACGTGGC	N3	2367/31128	51/154	5.97 × e^-20^
ZDNAFORMINGATCAB1	ATACGTGT	N3	771/31128	20/154	1.99 × e^-09^
T/GBOXATPIN2	AACGTG	N3	7495/31128	66/154	2.36 × e^-07^
DRECRTCOREAT	RCCGAC	N3	7166/31128	62/154	1.37 × e^-06^
SORLIP1AT	GCCAC	N3	15027/31128	101/154	1.11 × e^-05^

UP1ATMSD	GGCCCAWWW	N5	4460/31128	167/705	2.79 × e^-11^
	RRCCGTTA*	N5	1809/31128	88/705	2.31 × e^-11^
E2F1OSPCNA	GCGGGAAA	N5	676/31128	33/705	4.69 × e^-05^
E2FANTRNR	TTTCCCGC	N5	676/31128	33/705	4.69 × e^-05^
E2FCONSENSUS	WTTSSCSS	N5	8895/31128	237/705	1.98 × e^-06^

ACGTABREMOTIFA2OSEM	ACGTGKC	N8	4552/31128	32/89	5.09 × e^-07^
	RACCACAR*	N8	4187/31128	25/89	2.13 × e^-04^

DRECRTCOREAT	RCCGAC	N9	7166/31128	74/96	5.80 × e^-29^
ACGTABREMOTIFA2OSEM	ACGTGKC	N9	4552/31128	45/96	5.55 × e^-14^

ACGTABREMOTIFA2OSEM	ACGTGKC	N10	4552/31128	109/343	8.19 × e^-16^
TGACGTVMAMY	TGACGT	N10	6796/31128	109/343	1.28 × e^-05^
ABREATRD22	RYACGTGGYR	N10	747/31128	22/343	3.28 × e^-05^
	ACGCG*	N11	6880/31128	122/279	7.90 × e^-16^

ACGTABREMOTIFA2OSEM	ACGTGKC	N12	4552/31128	67/197	8.52 × e^-12^
DRECRTCOREAT	RCCGAC	N12	7166/31128	73/197	6.47 × e^-06^
ELRECOREPCRP1	TTGACC	N12	11015/31128	97/197	4.57 × e^-05^

ACGTABREMOTIFA2OSEM	ACGTGKC	N13	4552/31128	77/149	2.96 × e-26
ZDNAFORMINGATCAB1	ATACGTGT	N13	771/31128	13/149	9.60 × e^-05^

UP2ATMSD	AAACCCTA	N14	4947/31128	137/275	5.56 × e^-39^
UP1ATMSD	GGCCCAWWW	N14	4460/31128	82/275	3.53 × e^-11^
SITEIIATCYTC	TGGGCY	N14	12341/31128	164/275	1.79 × e^-11^

MYCATERD1	CATGTG	N15	11214/31128	89/171	1.36 × e^-05^
SORLREP3AT	TGTATATAT	N15	3065/31128	33/171	1.34 × e^-04^

UP1ATMSD	GGCCCAWWW	N18	4460/31128	193/430	2.38 × e^-52^
E2FCONSENSUS	WTTSSCSS	N18	8895/31128	160/430	6.74 × e^-05^
	RnGTGGGC*	N18	2046/31128	51/430	3.92 × e^-05^

ELRECOREPCRP1	TTGACC	N19	11015/31128	112/210	8.12 × e^-08^

ACGTABREMOTIFA2OSEM	ACGTGKC	N20	4552/31128	43/91	1.40 × e^-13^
LRENPCABE	ACGTGGCA	N20	1122/31128	12/91	1.07 × e^-04^

Motifs are according to the PLACE database [14]. *Motifs identified from a study in mammalian systems [89]. R, A or G; W, A or T; S, C or G; K, G or T; Y, C or T; n, any nucleotide.

#### Genes in upregulated clusters

The WB-BOX motif TTTGACT was identified in clusters N0, N11, and N19. Genes in clusters N0 and N19 were generally induced by abiotic stresses, whereas genes in cluster N11 were upregulated markedly in roots by salt treatment. The WB-BOX represents a binding site for WRKY transcription factors [70], which have 12, 4, and 5 members in clusters N0, N11, and N19, respectively. Among other clusters, only N12 included a number of WRKY factors (five in total). It seems that WRKYs correlate well with pathogen response activity. Genes in cluster N0 were also induced by osmotic and ionic stresses, and the additional HSF motif (heat shock factor binding) (A|G)GAANNTTC was over-represented in this cluster (with N representing any nucleotide). Also, two HSF transcription factors are included in this cluster.

Genes in clusters N1 and N8 were responsive to light treatment. The ABRE motif ACGTG(G|T)C was identified in both clusters, together with an unknown motif, namely (A|G)ACCACA(A|G). ACGTG(G|T)C is similar to the G-Box motif that mediates light signaling [12]. Also identified in cluster N1 was the I-Box motif GATAAG(A|G).

Clusters N3, N9, N10, N12, N13, and N20 were induced by ABA treatment to variable degrees. The ABRE motif was over-represented in these clusters, but only cluster N3 contained the ABRE-binding proteins AREB1 and AREB2 [13]. The DRE motif (A|G)CCGAC was identified in the clusters N3, N9, and N12, which is in agreement with the strong induction by cold stress of genes in these clusters [13]. Within these three clusters, four, two, and one DRE-binding (DREB) transcription factors were included, respectively. Although cluster N11 included seven DREB genes, the DRE motif was not over-represented in this cluster. Cluster N11 also contains an additional nine ERF/AP2 transcription factors. Clusters N3 and N9 additionally exhibited the EVENINGAT motif (AAAATATCT), which functions in the circadian control of gene expression [71]. Further identified were the P1BS motif (GNATATNC) and an unknown, hypothetical motif (A|G)(C|T)TAA(A|T)NNNTGA(C|T) in cluster N10, and the 2S-SEED-PROTBANAPA motif (CAAACAC) in cluster N13.

Over-representation of the well known ABRE motif in multiple clusters of genes that respond to either light or ABA treatment points toward the existence of additional motifs [13]. These could be the I-Box and DRE motifs that are over-represented in these clusters, and other putatively *cis*-acting motifs are suggested by the analysis. More likely, however, is the presence of transcriptional control mechanisms that act on *cis*-element binding proteins rather than on the promoter elements.

#### Genes in downregulated clusters

Motifs of prevalence similar to those in upregulated genes appear to be largely absent from the stress-repressed genes in clusters N4, N7, and N17. For cluster N15 genes, strongly downregulated by osmotic and high salinity stresses in roots, the MYCATERD1 motif (CATGTG) and the SORLRP3AT motif (TGTATATAT) were identified. Clusters N2, N5, N14, and N18 included many genes related to general gene expression functions, protein synthesis, cell organization, and metabolism. Several known motifs were enriched in these clusters. The UP1ATMSD motif GGCCCA(A|T)(A|T)(A|T), which is related to axillary bud growth [72], was over-represented in all four clusters. Additionally, over-represented in genes in cluster N2 were the I-Box motif GATAAG(A|G) and the SORLIP5AT motif GAGTGAG [73], which appear to be connected to the expression of genes in metabolic functions. Cluster N5 showed the E2F1OSPCNA motif GCGGGAAA, the E2FANTRNR motif TTTCCCGC, and the E2FCONSENSUS motif (A|T)TT(G|C)C(G|C)(G|C). These motifs are typically associated with genes that are involved in cell cycle progression and cell division [74-76]. At lower frequency, clusters 14 and 18 exhibited similar motif combinations (Table 2).

**Figure 4:**
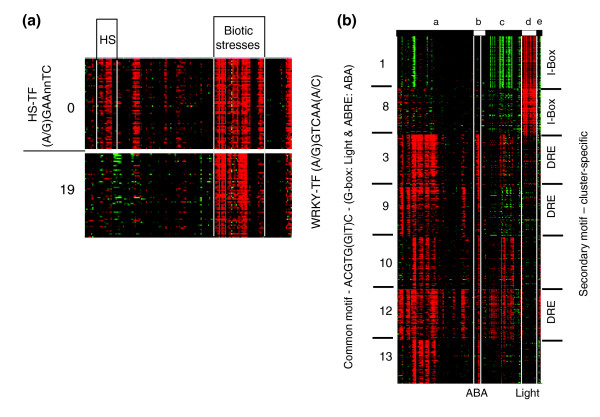
Conserved or cluster-specific *cis*-elements. **(a) **Motifs identified in clusters N1, N8, N3, N9, N10, N12, and N13. These clusters were induced by light or ABA. A common motif ABRE united genes within these clusters. Additional motifs that distinguish genes in individual clusters are included (I-Box or DRE). See Table 2 for identified motifs. **(b) **5'-regulatory motifs identified from clusters N0 and N19. The WRKY motif was identified in both clusters, whereas the HSF motif was present only in cluster N0.

In general, fuzzy k-means clustering applied to 5'-regulatory sequences confirmed known motifs in the major stress-responding clusters, whereas different clusters shared subsets of these motifs. Additional, secondary motifs between and within large clusters are suggested (Figure 4b, Table 2), but attempts to distinguish between clusters that shared similar expression patterns through motif analyses alone proved inconclusive. Different approaches will be necessary to reveal how combinations of motifs may control gene expression. Methods for identifying such combinations are emerging [77].

### Integrating AtGenExpress and *Arabidopsis *root transcript profiles

**Figure 5:**
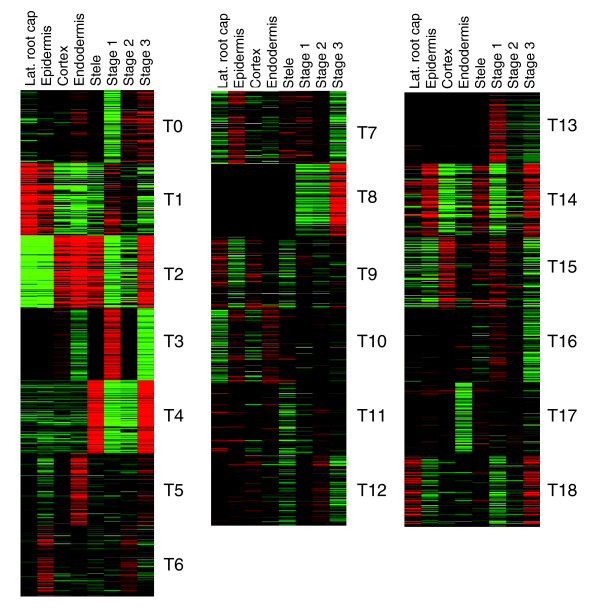
Tissue-specific characteristics of abiotic stress-regulated transcripts. The expression of 12,360 transcripts in different tissues, cell lineages, and at three developmental stages in *Arabidopsis *root [19], separated into 19 clusters.

Very few data are available to date that correlate stress-related transcript changes and cell-specific or tissue-specific gene expression. We focused on the tissue-specific response to stress in detail by including a dataset in which cell type-specific and growth stage-specific gene expression in *Arabidopsis *roots was recorded [19]. Among the probes printed on the Affymetrix chip, 12,360 were considered present in at least one of the three developmental stages of the root. These stages identify genes in cell division and early root expansion growth (stage 1), the region of maximum elongation growth (stage 2), and genes in the root maturation region (stage 3). Also recorded was the gene expression pattern in different cell lineages: the lateral root cap, epidermis, cortex, endodermis, and in the vasculature (stele). Here, intensity values were compared for each gene in the three developmental stages and in each cell lineage against its average intensity across all stages or cell lineages, and the difference in expression provided a measure of cell specificity and stage specificity for each gene. Fuzzy-k means clustering revealed a clear pattern for the 12,360 genes in the root dataset, which separated into 19 clusters (T0 to T18; Figure 5). For example, genes in cluster T2 were more highly expressed in the cortex, endodermis, and stele during developmental stage 3, identifying mature regions of the root. In contrast, genes in cluster T3 were highly expressed during stage 1 development, and present at lower level in stage 3 regions of the root and in the endodermis. Cluster T4 shows genes with strong expression in the stele during stage 3.

**Figure 6:**
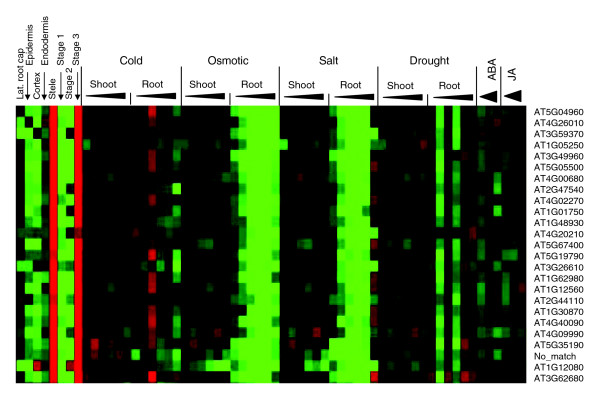
Stress down-regulated genes highly expressed in Stele during stage 3 development. AT5G04960, pectinesterase family protein; AT4G26010, peroxidase, putative, peroxidase ATP13a; AT3G59370, expressed protein; AT1G05250, peroxidase, putative, similar to peroxidase ATP11a; AT3G49960, peroxidase, putative, identical to peroxidase ATP21a; AT5G05500, pollen Ole e1 allergen and extensin family protein; AT4G00680, actin-depolymerizing factor, putative; AT2G47540, pollen Ole e1 allergen and extensin family protein; AT4G02270, pollen Ole e1 allergen and extensin family protein; AT1G01750, actin-depolymerizing factor, putative; AT1G48930, endo-1,4-beta-glucanase, putative cellulase; AT4G20210, terpene synthase/cyclase family protein; AT5G67400, peroxidase 73 (*PER73*) (P73) (*PRXR11*); AT5G19790, encodes a member of the ERF (ethylene response factor) subfamily B-6 of ERF/AP2 transcription factor family (*RAP2.11*); AT3G26610, polygalacturonase, putative pectinase; AT1G62980, expansin, putative (*EXP18*); AT1G12560, expansin, putative (*EXP7*); AT2G44110, seven transmembrane MLO family protein/MLO-like protein 15 (*MLO15*); AT1G30870, cationic peroxidase, putative; AT4G40090, arabinogalactan-protein (*AGP3*); AT4G09990, expressed protein; AT5G35190, proline-rich extensin-like family protein; No match, no match; AT1G12080, expressed protein; AT3G62680, proline-rich family protein.

**Figure 7:**
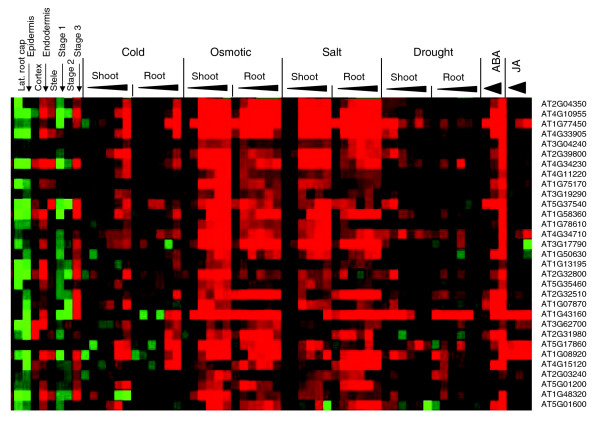
Stress and ABA upregulated genes in mature roots are also expressed in shoots. AT2G04350, long-chain-fatty-acid-CoA ligase family protein; AT4G10955, lipase class 3 family protein; AT1G77450, no apical meristem (NAM) family protein, similar to GRAB1; AT4G33905, peroxisomal membrane protein 22 kDa; AT3G04240, O-linked N-acetyl glucosamine transferase; AT2G39800, delta 1-pyrroline-5-carboxylate synthetase A/P5CS A (*P5CS1*); AT4G34230, cinnamyl-alcohol dehydrogenase, putative; AT4G11220, reticulon family protein (*RTNLB2*); AT1G75170, SEC14 cytosolic factor family protein/phosphoglyceride transfer family; AT3G19290, ABA-responsive element-binding protein 2 (*AREB2*); AT5G37540, aspartyl protease family protein, weak similarity to CND41; AT1G58360, amino acid permease I (*AAP1*); AT1G78610, mechanosensitive ion channel domain-containing protein; AT4G34710, arginine decarboxylase 2 (*SPE2*); AT3G17790, acid phosphatase type 5 (*ACP5*); AT1G50630, expressed protein; AT1G13195, zinc finger (C3HC4-type RING finger) family protein; AT2G32800, protein kinase family protein, contains dual protein kinase domains; AT5G35460, expressed protein; AT2G32510, protein kinase family protein, contains protein kinase domain; AT1G07870, protein kinase family protein, contains protein kinase domain; AT1G43160, encodes a member of the ERF (ethylene response factor) subfamily B-4 of ERF/AP2 transcription factor family (RAP2.6); AT3G62700, glutathione-conjugate transporter, putative, similar to AtMRP4; AT2G31980, cysteine proteinase inhibitor-related; AT5G17860, cation exchanger, putative (*CAX7*), similar to NKX3_HUMAN sodium/potassium/calcium exchanger 3 precursor; AT1G08920, sugar transporter, putative, similar to ERD6 protein (*Arabidopsis thaliana*); AT4G15120, VQ, motif-containing protein; AT2G03240, EXS family protein/ERD1/XPR1/SYG1 family protein, similar to PHO1; AT5G01200, myb family transcription factor; AT1G48320, thioesterase family protein; AT5G01600, ferritin 1 (*FER1*).

Among the 12,360 genes recognized in roots, 5,963 exhibited significant membership values in the stress expression profiles, and these genes were further analyzed. The intersections between tissue and stress clusters are shown in Table 3, revealing specific reactions to different stress conditions in distinct cell lineages and developmental stages of the *Arabidopsis *root. The nature of the genes at the intersection between cell specificity/development in roots and stresses (Table 3) is detailed in Additional data file 9. In the following discussion, we address stress-regulated genes within the context of their expression in a developmental and cell-specific context. Examples highlight root-specific genes that are downregulated by abiotic stress and that are highly expressed in root cap and epidermis of stage 1 roots under optimal conditions (Figure 6), and genes that are upregulated by stress and that, under nonstress conditions, are highly expressed in the vasculature (stele), endodermis, and cortex in stage 3 roots (Figure 7).

**Table 3:** Intersection between tissue-specific expression and stress responses

		Cluster (analysis of different stress conditions; N0 to N19)																			
		
		0	1	2	3	4	5	6	7	8	9	10	11	12	13	14	15	16	17	18	19
Clusters (according to tissue specificity)	0	36*	3	53	8	1	38*	0	2	7	4	32*	3	5	4	0	13	2	19*	4	12
	1	42*	5	26	9	2	7	0	6	2	3	12	5	12	3	0	28*	2	6	2	23*
	2	50*	13	56	14	3	21	0	5	12	9	26*	9	4	11	0	17	0	10	0	19*
	3	3	22*	251*	1	0	234*	0	0	2	0	4	1	3	1	243*	2	3	5	336*	5
	4	9	4	31	6	18*	30	0	28*	1	9	13	5	4	10	0	16	3	2	0	5
	5	110*	11	18	6	0	1	0	2	10	5	56*	16	19	13	0	0	1	26*	0	7
	6	13	3	32	2	0	49*	0	0	4	1	10	3	1	2	1	3	3	10	12	3
	7	3	1	36	2	0	55*	0	0	0	1	2	0	1	0	10	0	0	4	41*	2
	8	21	6	8	7	15*	1	0	3	4	1	10	4	12	9	0	20*	0	8	0	8
	9	25	12	21	5	0	4	0	0	5	6	7	11	24*	1	0	0	1	5	0	4
	10	13	13	25	3	1	17	0	0	4	1	26*	1	3	2	0	1	0	13	4	4
	11	50*	1	15	4	0	7	0	0	3	2	25*	4	4	5	1	1	2	12	0	12
	12	2	6	52	3	0	32*	0	0	0	2	3	1	1	3	1	2	0	1	5	3
	13	8	26*	94*	3	0	35*	0	0	1	4	11	3	2	2	3	5	3	3	1	1
	14	19	1	8	11	1	5	0	7	2	5	16	2	0	4	0	9	0	5	0	11
	15	1	15	58	4	0	25	0	0	1	2	3	2	1	0	4	1	0	4	3	2
	16	6	10	61	1	0	20	0	0	2	0	3	1	0	2	5	1	2	3	6	0
	17	2	3	18	4	0	30	0	0	0	3	0	0	5	0	4	0	1	3	12	1
	18	56*	1	5	2	0	1	0	1	4	6	8	5	18	3	0	2	2	4	0	12
	Sum	469	156	868	95	41	612	0	54	64	64	267	76	119	75	272	121	25	143	426	134

Shown are numbers of genes at intersections between clusters of tissue-specific expression patterns (T0 to T18) in roots and clusters emerging from the analysis of stress treatments (N0 to N19). *Gene-enriched intersections.

#### Stress down-regulated genes in roots

Three different regulatory patterns for downregulated genes emerged. First, the stress clusters N14 and N18 included mainly genes related to the key machineries of gene expression and protein synthesis, most of which organized in tissue cluster T3 (stage 1 specific) and T7 (stages 1 and 2). Cluster N18 includes genes that mainly function in protein synthesis: more than 190 ribosomal proteins, 5 tRNA synthetases, 13 translation initiation/elongation proteins, 10 chaperonin proteins, and a few genes related to lysine or arginine synthesis. Also included in N18 were DNA replication licensing factors, nucleosome assembly proteins, histones H2A and H3, small nuclear ribonucleoproteins, and signaling G proteins. Several GO categories are enriched in cluster N18: GO:0042254 (ribosome biogenesis and assembly), GO:0043037 (translation), GO:0015450 (protein translocase activity), GO:0046112 (nucleobase biosynthesis), GO:0006333 (chromatin assembly), and GO:0006525 (arginine metabolism). In contrast, cluster N14 collected genes mainly related to transcription, such as 12 DEAD/DEAH box helicases and 11 polymerases (or similar to polymerase functions), with a few genes involved in protein synthesis functions. Several GO categories were over-represented in N14: GO:0016072 (rRNA metabolism), GO:0003899 (DNA-directed RNA polymerase activity), GO:0004527 (exonuclease activity), GO:0008026 (ATP-dependent helicase activity), GO:0042254 (ribosome biogenesis and assembly), and GO:0006396 (RNA processing). Cluster N14 was different from N18 in that N14 was slightly induced by cold stress but N18 was not. The expression of genes in these two clusters can most parsimoniously be rationalized by developmental regulation.

Second, the stress clusters N4 and N7 contained genes that were placed mainly in the tissue clusters T4, T8, and T14, all of which represent stage 3 specific genes. Interestingly, among the downregulated genes were those related to cell wall modification that are specifically expressed in the stele (T4 and T14), such as expansins, extensins, (putative) cellulases, pectinesterases, and peroxidases. Enriched in these two clusters were GO:0006979 (response to oxidative stress), GO:0007047 (cell wall organization and biogenesis), GO:0009653 (morphogenesis), GO:0042545 (cell wall modification), GO:0010054 (trichoblast differentiation), and GO:0005516 (calmodulin binding). Together, these clusters appear to identify the portion of the transcriptome that controls root maturation, which is downregulated under stress treatments of the root system.

Finally, genes in the stress clusters N2 and N5 represented a combination of the previously discussed patterns. These clusters included genes regulated developmentally (in the tissue clusters T3, T6, T7, T12, T13, T15, and T16 [stage 1 or stage 2 specific]), and genes downregulated by stress signaling (mainly in the tissue clusters T0, T1, T2, or T4). Over-represented in cluster N2 were genes involved in metabolism, which included amino acid, cell wall, carbohydrate, lipid, nucleotide, and secondary metabolism biosynthetic functions. The following GO categories were over-represented: GO:0043038 (amino acid activation), GO:0009658 (chloroplast organization and biogenesis), GO:0006779 (porphyrin biosynthesis), GO:0019321 (pentose metabolism), GO:0004312 (fatty-acid synthase activity), GO:0006769 (nicotinamide metabolism), GO:0005528 (FK506 binding), GO:0016117 (carotenoid biosynthesis), GO:0015994 (chlorophyll metabolism), and GO:0009606 (tropism). Genes related to DNA synthesis, chromatin structure, cell cycle, and cell division were abundant in downregulated cluster N5; the following were over-represented: GO:0006260 (DNA replication), GO:0007049 (cell cycle), GO:0000910 (cytokinesis), GO:0007010 (cytoskeleton organization and biogenesis), and GO:0016071 (mRNA metabolism). Cluster N5 also includes some genes related to metabolic processes, as indicated the over-represented GO categories GO:0009853 (photorespiration), GO:0019758 (glycosinolate biosynthesis), GO:0044272 (sulfur compound biosynthesis), and GO:0009067 (aspartate family amino acid biosynthesis).

In essence, the genes downregulated by different stresses are expressed under ideal growth conditions close to the root meristem and in the region of strongest cell expansion. Furthermore, genes related to the functional categories of mRNA and protein synthesis, cell cycle control, and primary metabolism categories were separated into differentially repressed clusters. This indicated active regulatory processes, other than a passive repression brought about merely by a general stressed physiologic state.

#### Stress upregulated genes in roots

A significant difference emerged when genes in stress upregulated clusters were viewed in their tissue-specific or cell-specific context. In the majority, these genes exhibited a high expression level at stage 3 (tissue clusters T0, T2, T5, T14, and T17) or high expression in root cap cells (T1 and T9). Because genes in these tissue clusters appeared with insignificant membership values only in the repressed clusters N14 and N18, we consider them representative of first responders to stress signaling. It appeared significant that these genes were upregulated in cells in the more mature region of the root, coinciding with the region of beginning root hair development.

Merging stress and tissue/cell specificity, a framework became recognizable. Genes in tissue clusters T3 and T7 were significantly downregulated during abiotic stress, and genes in tissue clusters T0, T1, T2, and T4 were either upregulated or downregulated under different stress conditions. In contrast, genes in tissue clusters T5 and T18 were mainly upregulated upon stress. Of particular importance may be the behavior of the genes in clusters T5 and T18. T5 genes were specifically expressed in endodermis cells in stage 3, whereas T18 genes exhibited high expression level in lateral root cap cells.

#### Cell lineage-specific and development-dependent stress response pathways

Focusing on abiotic stresses alone (cold, osmotic, salinity, drought, and the hormones ABA and JA), the 12,360 probes present in roots were analyzed by fuzzy k-means clustering. The analysis of this smaller set of treatments separated the genes into 66 clusters (Additional data file 10). Intersections of stress specificity and spatial or temporal expression characteristics are illustrated by two examples.

Figure 6 shows root-expressed genes that are strongly downregulated predominantly during osmotic and salt stresses. The identity of the genes with high expression in the stele of stage 3 roots highlights functions that are associated with the decline of root growth. Abundantly represented were peroxidases, extensins, and PRP-like proteins, and functionally unknown proteins. A contrasting behavior is shown in Figure 7, which identifies a cluster with osmotic and salt stress upregulated genes. These genes are uniformly upregulated by ABA and, in part by JA, while ABA upregulation generally also extends into the shoots. This cluster includes many transcripts for functions in signaling and transport, and a number of genes that have been well characterized, such as transcripts for proline synthesis, glutathionine-conjugate transport, ferritin, calcineurin phosphoesterase, SEC14, and the ABA-responsive AREB2. The complete set of data is included in the Additional data file 10.

## Conclusion

Integration of diverse, large-scale datasets into a framework that then describes and explains the functioning of an organism remains an elusive goal of genomics-type approaches. Combining three types of data, we analyzed in context the genome-wide expression profiles modulated by a number of stress conditions, regulatory *cis*-elements in promoters, and cell-specific and developmental age-specific root transcripts and their reaction to stress in the model crucifer *Arabidopsis thaliana*. A recent analysis used the AtGenExpress dataset by focusing on responses under nine experimental conditions and identified similarities between conditions [78], whereas our approach distinguished similarities and differences between genes under all conditions. The fuzzy k-means clustering tool [16] generated reliable clustering results because known stress response genes, originally reported in single-gene analyses, were generally confirmed by their inclusion in appropriate clusters [3,4,20,79]. The tool provided flexibility to arrive at realistic cluster structures that could be adjusted by the choice of different membership values to take into account data from different sources.

Detailed analysis focused on cluster N12, which included genes responsive to most environmental perturbations. This type of analysis is similar to that in a study that identified cellular stress response genes in yeast from global transcript profiles of stress responses [80]. In terms of functional categories a significant overlap is evident, although the yeast analysis identified a larger number of genes involved in carbohydrate metabolism in this group of common stress genes compared with the *Arabidopsis *list. Many of the genes in our analysis encode stress-responsive functions in animals and yeasts, such as the Snf1 kinase-related, phoshoinositol-related, and Bax-inhibitor related pathways. They may represent the evolutionarily conserved cellular stress response, originating from damage recognition of, for example, lipid membranes, proteins, or DNA, and mediated by signals related to calcium and ROS [11,81]. In plants, signals may also be communicated by ethylene [82] and are largely independent of ABA. Although responding to many stresses, ROS and ethylene signaling cannot act as a systemic coordinator of gene expression in the way that this is accomplished by ABA.

Hypothetically, ROS [81] or ethylene induce signaling mainly locally in stress responses, and the genes in cluster N12 appear to elicit local responses but have no function in long distance communication. In agreement with this hypothesis, no cluster specific for ACC treatment emerged, and neither was a correlation between ethylene treatments and the stress cluster structure identified in fuzzy k-means analysis [31].

The correlation of a number of previously studied 5'-regulatory, *cis*-acting sequences with particular stress conditions, biotic and abiotic alike, was confirmed [12,13,70], and the presence of additional 5'-regulatory response elements was identified (Table 2). The ABRE motif ACGTG(G|T)C was over-represented in multiple clusters responding to either light or ABA treatment, indicating that the motif is essential but not sufficient to explain the multiplicity of clusters. Secondary motifs that modify ABA responsiveness are identified. Within these clusters, the I-Box and DRE motifs emerged and others are strongly suggested, although detailed studies have not been conducted on these putatively novel regulatory elements. Another motif, the W-box, was over-represented in several clusters induced by biotic stresses, and the corresponding W-box binding transcription factors, namely WRKYs, were themselves included in these clusters.

The chosen way to integrate datasets revealed relationships between stress regulation and tissue-specific expression in the *Arabidopsis *root. In particular, the downregulation of genes during osmotic challenge and, although moderately, by ABA in roots identified genes that, under nonstress conditions, are highly expressed in cells of the vascular tissue and in the mature root (Figure 6). The stress-repressed genes in these clusters are responsible for the physiologic effects of stress that result in impeded growth; most of these genes reflect metabolic pathways and functions that signal injury and challenges to organ integrity. In stark contrast, other clusters identified genes that are upregulated by ABA, in part also by JA, and upregulation is not solely confined to the roots (Figure 7). Cell specificity is less pronounced in these clusters but the genes included tend to be more highly expressed in cells of the cortex, endodermis, and vasculature in mature regions of roots. Genes with known and conjectured signaling functions dominate in these clusters. This findings appears to implicate the endodermis and stele of mature roots as playing critical roles in counteracting the effects of many stresses. Included are many unknown genes, whose functions in environmental stress protection have not yet been analyzed.

Our approach represents one way to integrate diverse, independent datasets to enhance understanding of the plant environmental stress transcriptome. Irrespective of the many experimental conditions, the analysis identified many genes that had previously been implicated in plant stress responses in detailed studies that focused on individual genes. In addition, the overall structure of 5'-regulatory sequences that resulted from this study corresponded to the results of other studies, but they also suggested the existence of additional putative *cis*-elements, which await detailed analysis. The clustering that emerged provides an interpretation for the interdependence and distinction of biotic and abiotic stress factors. It defines an evolutionarily conserved basic set of stress response genes. Genes related to ROS-generating and ROS-detoxifying functions and ethylene action were scattered in virtually all major clusters, which appears to indicate the fundamental roles that these proteins play in diverse sensing and signaling pathways. Finally, the correlation of changes in transcript abundance and the spatial and temporal resolution of expression patterns in *Arabidopsis *roots add a new dimension. The predictions intrinsic in the cluster structures and their gene compositions are models that should be helpful in designing more detailed analyses.

## Materials and methods

### Affymetrix microarray data

Transcript profiles that reflect responses of *Arabidopsis *to different abiotic stress conditions were obtained from Weigel World [83], which had been processed via gcRMA [84]. For biotic stresses, hormones, different light regimens, and chemicals (t-zeatin, tri-iodobenzoic acid, AgNO_3_, and cycloheximide) treatments, the CEL files of the Affymetrix ATH1 microarray data were downloaded from TAIR [85], and processed into expression estimates using the gcRMA method with default settings implemented in Bioconductor [86]. For each experiment, the log2 intensities of individual probe sets were averaged across two replicates for both treatments and control, and their differences were used as log2 values of fold changes (treatments/control). Details of the treatment conditions, excerpted from the AtGenExpress project, are included in Additional data file 1, and the processed data are listed in Additional data file 2.

The microarray data pertaining to cell type-specific and growth stage-specific gene expression in *Arabidopsis *roots have previously been described [19]. The CEL files for these data were downloaded from the *Arabidopsis *Gene Expression Database [18] and processed into expression estimates as described above. The log2 intensities of every individual probe sets were averaged across three replicates for cell type-specific profiles, or four replicates for stage-specific profiles (Additional data file 6). MAS 5.0, which calls as present or absent each probe set in each slide, was calculated using the 'affy' package implemented in Bioconductor. Only probe sets with calls of present in all four replicates from at least one of the stage samples were analyzed. Excluded were 356 genes that had been shown to be induced by protoplasting of root cells [19]. The remaining 12,360 probes were analyzed.

### Clustering analysis

The analysis of stress, hormone, chemical, and light treatments was similar to the procedure described previously [21]. The log2 fold change values (treatment/control) of entire probe sets were analyzed with fuzzy k-means clustering [16]. The parameter was set as k = 300 for the complete set of treatments. In the most economical manner, 180 centroids were identified, and clustered such that any probe was assigned to the cluster in which it had highest membership value (Additional data file 3). By applying a cutoff of 0.035, 10,671 probes were separated into these clusters. For each gene, the sum of its membership values with the 180 centroids is 1. Therefore, the average membership value is 0.006. We considered 0.035, around six times higher than the average value, to be a significant cutoff.

To reveal the cell type-specific and stage-specific gene expression patterns, the relative expression value for each probe in each cell type or stage was calculated, by subtracting the probe's average intensity across the cell type samples or the stage samples from its intensity in that cell type or stage samples (Additional data file 7). The relative expression values were assembled and analyzed by fuzzy k-means, with the parameter k set at 30. Nineteen well defined clusters were recovered, and no cutoff values were applied (Figure 5 and Additional data file 8).

To focus on abiotic stress responses, we selected datasets for cold, osmotic, salt and drought stresses, and the hormones ABA and JA, for those probes present in roots. These were clustered separately, as previously, and resulted in 66 clusters. The corresponding *Arabidopsis *gene locus for each probe set followed the annotation by TAIR [85].

### Differentially expressed genes

The limma method implemented in Bioconductor was used to identify differentially expressed genes [22]. The original expression datasets from all conditions, derived from gcRMA, were used to construct the linear model. Different contrast matrices were utilized to identify the genes that were differentially expressed under at least one condition/time point among all conditions, or among the time course treatments of cold, osmotic, salinity, wounding, or pathogen treatments.

### Gene Ontology analysis

The Clench 2.0 program [87] was used to identify over-represented GO categories within a group of genes.

### Motif analysis

The motifs listed in the PLACE database were collected [14]. Their frequencies of appearance in the promoter regions (1,000 base pairs upstream of the coding region, downloaded from TAIR) of all genes in the entire genome were scanned using the patmatch program [88]. For each motif, its frequency of appearance in any cluster was compared with its frequency in all promoters predicted for the entire genome. A *P *value was calculated based on hypergeometric distribution [69]:

p(m)=∑l=mmin⁡(k,M)(kl)×(K−kM−l)(KM)
 MathType@MTEF@5@5@+=feaafiart1ev1aaatCvAUfeBSjuyZL2yd9gzLbvyNv2Caerbhv2BYDwAHbqedmvETj2BSbqee0evGueE0jxyaibaiKI8=vI8tuQ8FMI8Gi=hEeeu0xXdbba9frFj0=OqFfea0dXdd9vqai=hGuQ8kuc9pgc9s8qqaq=dirpe0xb9q8qiLsFr0=vr0=vr0dc8meaabaqaciGacaGaaeqabaqadeqadaaakeaacaWGWbGaaiikaiaad2gacaGGPaGaeyypa0ZaaabCaeaadaWcaaqaamaabmaaeaqabeaacaWGRbaabaGaamiBaaaacaGLOaGaayzkaaGaey41aq7aaeWaaqaabeqaaiaadUeacqGHsislcaWGRbaabaGaamytaiabgkHiTiaadYgaaaGaayjkaiaawMcaaaqaamaabmaaeaqabeaacaWGlbaabaGaamytaaaacaGLOaGaayzkaaaaaaWcbaGaamiBaiabg2da9iaad2gaaeaaciGGTbGaaiyAaiaac6gacaGGOaGaam4AaiaacYcacaWGnbGaaiykaaqdcqGHris5aaaa@52F2@

where *M *is the number of promoters within the cluster, *m *is the number of promoters within the cluster that contain the motif, *K *is the total number of promoters in the genome, and *k *is the total number of the promoters in the genome that contain the motif.

Over-represented motifs within clusters were identified by their *P *values. Also included in the analysis was a list of *cis*-elements identified from a study conducted in mammalian systems [89].

## Additional data files

The following additional data are available with the online version of this paper. Additional data file 1 includes the microarray datasets used for this analysis. Additional data file 2 includes the stress datasets used for fuzzy k-means analysis. Additional data file 3 includes clustering results of all stress datasets. Additional data file 4 includes a comparison between clusters N6 and N53. Additional data file 5 shows all genes in the common stresses response cluster N12. Additional data file 6 provides original data of gene expression in roots. Additional data file 7 shows processed root dataset used for fuzzy k-means analysis. Additional data file 8 shows clustering results for the root dataset. Additional data file 9 shows the intersection between stress clustering and root clustering. Additional data file 10 shows clustering results for abiotic stresses in roots.

## Supplementary Material

Additional data file 1 Microarray datasets used for this analysis, including the descriptions of the treatments and conditions. The data come from AtGenExpress (abiotic and biotic stresses, elicitor treatments, hormone treatments, organ-specific expression), and transcription data in different cell lineages and developmental stages of the root. Click here for file

Additional data file 2 Stress datasets used for Fuzzy K-means analysis. Click here for file

Additional data file 3 Clustering results of all stress datasets. Click here for file

Additional data file 4 Comparison between clusters N6 and N53 (legend as in Figure 2). Click here for file

Additional data file 5 All genes in the common stresses response cluster N12. Click here for file

Additional data file 6 Original data for gene expression in roots. Click here for file

Additional data file 7 Processed root dataset used for fuzzy k-means analysis. Click here for file

Additional data file 8 Clustering results for the root dataset. Click here for file

Additional data file 9 Intersection between stress clustering and root clustering. Click here for file

Additional data file 10 Clustering results for abiotic stresses in roots. Click here for file
