# Supplementary material for: Integration of Arabidopsis thaliana stress-related transcript profiles, promoter structures, and cell-specific expression
Source: Genome Biol. 2007 Apr 4;8(4):R49. doi: 10.1186/gb-2007-8-4-r49 (PMC1896000; doi:10.1186/gb-2007-8-4-r49)
Supplement: Additional data file 1 — Microarray datasets used for this analysis, including the descriptions of the treatments and conditions. The data come from AtGenExpress (abiotic and biotic stresses, elicitor treatments, hormone treatments, organ-specific expression), and transcription data in different cell lineages and developmental stages of the root. [file gb-2007-8-4-r49-S1.pdf]

	Treatment (or Tissue)	Treatment Conditions	Number of Time Points	TAIR Submission Number
Abiotic Stresses (from Weigle World [83])				
	Cold	4 ^{o}C	12	ME00325
	Osmotic	300 mM Mannitol	12	ME00327
	Salt	150 mM Salt	12	ME00328
	Drought	15 min. dry air stream	12	ME00338
	Oxidative	10 μm Methyl Viologen	12	ME00340
	Wounding	Punctured with pins	14	ME00330
Hormone (from TAIR [85])				
	ABA	10 μM ABA	3	ME00333
	1-Aminocyclopropane-1-carboxylic Acid (ACC)	10 μM ACC	3	ME00334
	Methyl Jasmonate (MeJA)	10 μM MeJA	3	ME00337
Elicitors (from TAIR [85])				
	Ca	1mM $CaCl_2$ + 2.5 mM $MgCl_2$	2	ME00332
	GST	1 μM GST	2	
	Hairpin Z (hrpz)	10 μM hrpz	2	
	GST-Necrosis-Inducing Phytophtora Protein 1 (npp)	1 μM npp	2	
	Flagellin (flg)	1 μM Flg22	2	
	Lipopolysaccharide (lps)	100 μg/ml LPS	2	
Biotic Stresses (from TAIR [85])				
	PstDC 3000	*Pseudomonas syringae* pv. tomato DC3000 10e8 cfu/ml	3	ME00331
	Pstavrrpm	*Pseudomonas syringae* pv. tomato avrRpm1 10e8 cfu/ml	3	
	Psthrcc	*Pseudomonas syringae* pv. tomato DC3000 hrcC- 10e8 cfu/ml	3	
	Pstpshp	*Pseudomonas syringae* pv. Phaseolicola 10e8 cfu/ml	3	
	Botrytis	*Botrytis cinerea* 5e5 spores/ml	2	ME00341
	E. orontii	*Erysiphe orontii*	6	ME00354
	Phytophthora	*Phytophthora infestans* 1e6 spores/ml	3	ME00342
Light Treatments (from TAIR [85])				
	AL, AS	UV-A light	2	ME00345
	BL, BS	blue light	2	
	FL, FS	far-red light	2	
	PL, PS	red light (pulse)	2	
	RL, RS	red light	2	
	UL, US	UV-A/B light	2	
	WL, WS	white light	2	

Supplemental Table 1 Continued

	Treatment (or Tissue)	Treatment Conditions	Number of Time Points	TAIR Submission Number
Chemicals (from TAIR [85])				
	ARR22-ox t-zeatin	10 μM t-zeatin on ARR22-ox plants	1	ME00356
	2,3,5-triiodobenzoic acid	10 μM 2,3,5-triiodobenzoic acid	1	ME00358
	$AgNO_3$	10 μM $AgNO_3$	1	ME00360
	cycloheximide	10 μM cycloheximide	1	ME00361
Root Data Set (from AREX [18, 19])				
	Lateral Root Cap		1	
	Epidermis		1	
	Ground tissue		1	
	Endodermis		1	
	Stele		1	
	Stage1		1	
	Stage2		1	
	Stage3		1	

Note:

For more details of the treatments, please refer to the AtGenExpress webpage at TAIR [85] or AREX [18, 19].
