# Supplementary figures and images for: Integration of Arabidopsis thaliana stress-related transcript profiles, promoter structures, and cell-specific expression
Source: Genome Biol. 2007 Apr 4;8(4):R49. doi: 10.1186/gb-2007-8-4-r49 (PMC1896000; doi:10.1186/gb-2007-8-4-r49)

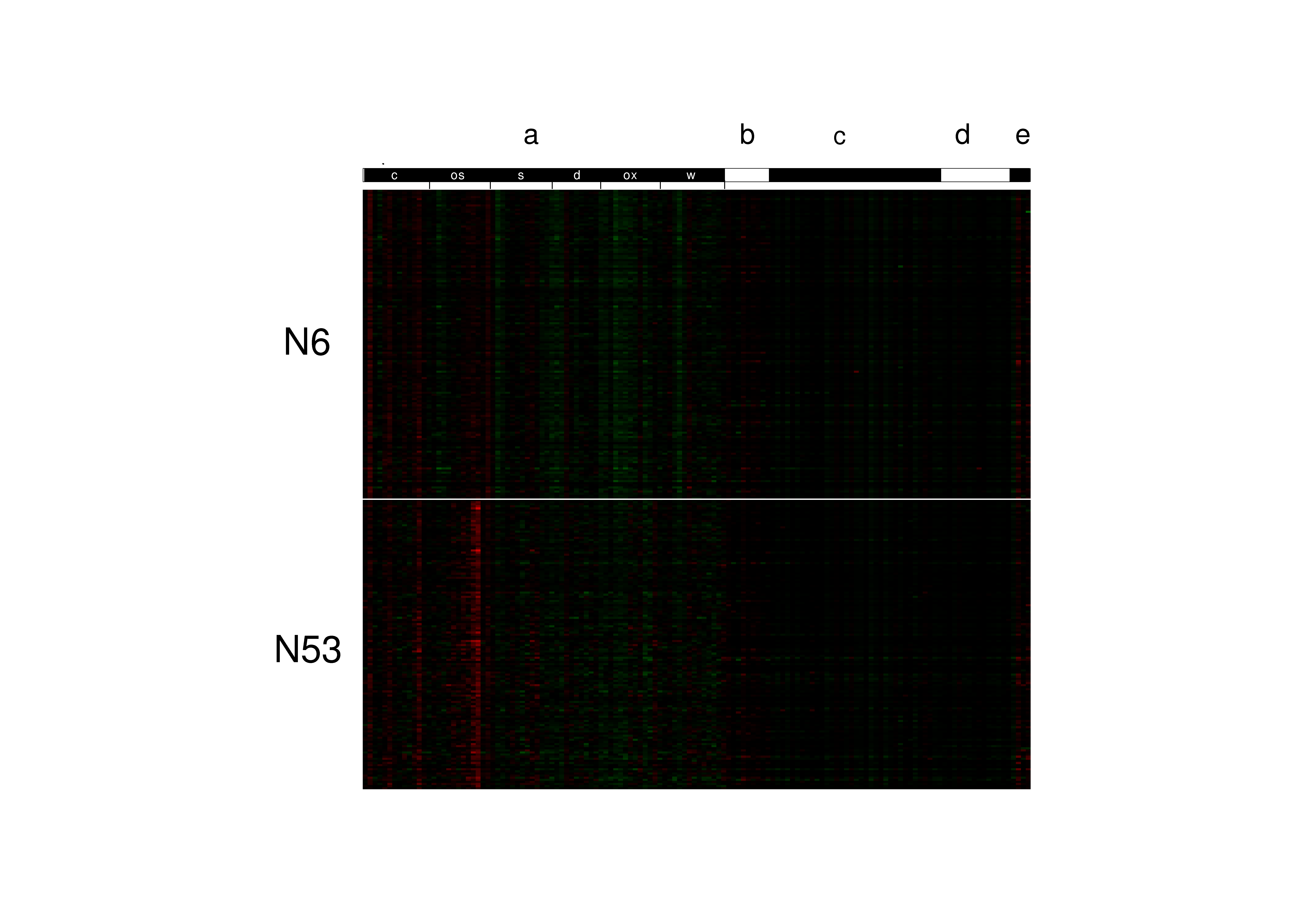

Supplement: Additional data file 4 — Comparison between clusters N6 and N53 (legend as in Figure 2). [file gb-2007-8-4-r49-S4.png]
